# Challenging the long-held “pied piper” hypothesis: evidence of southward migration of corn earworm (*Helicoverpa zea*) in North America

**DOI:** 10.3389/finsc.2026.1726127

**Published:** 2026-04-24

**Authors:** Amina A. Twaibu, Eduardo S. Calixto, Julien M. Beuzelin, Isaac L. Esquivel, Silvana V. Paula-Moraes

**Affiliations:** 1 West Florida Research and Education Center, University of Florida, Jay, FL, United States; 2 Everglades Research and Education Center, University of Florida, Belle Glade, FL, United States; 3 North Florida Research and Education Center, University of Florida, Quincy, FL, United States; 4 Department of Entomology, University of Nebraska, Lincoln, NE, United States

**Keywords:** bidirectional migration, insect resistance management, integrated pest management, movement ecology, stable hydrogen isotopes

## Abstract

Migration plays an important role in the ecology of insect pests, including *Helicoverpa zea*, a major pest of multiple crops across the U.S. The long-standing “pied piper” hypothesis proposes that *H. zea* populations migrate northward each summer to exploit seasonal resources but because cannot overwinter in the lethal winter conditions, populations of this pest fail to continue to be established in higher latitudes in North America. This study investigated the natal origins and migratory connectivity of *H. zea* moths collected across Florida between 2017 and 2024 using stable hydrogen isotope analysis of wing tissues. Through this approach, we traced the origins of 249 individuals, revealing that most late-season moths originated locally or from southern U.S. regions. However, a distinct subset exhibited isotopic signatures indicative of long-distance migration from northern areas, including the upper Midwest and Corn Belt. Estimated flight distances for these northern migrants ranged from 1,375 to 1,725 km, with dispersal patterns showing a predominant northwest-to-southeast orientation. These findings provide evidence that *H. zea* populations engage in bidirectional migration. This *H. zea* reverse migration has critical implications for integrated pest management (IPM) and insect resistance management (IRM) to insecticides and Bt traits, considering the risk of gene flow of populations under continuous selection for resistance in both Cotton and Corn Belts.

## 1 Introduction

Migration plays a critical role in insect pest ecology, influencing population dynamics, gene flow, and ecosystem processes across broad geographic scales (1–4). By engaging in long-distance movements, insect pests contribute to regional connectivity, transporting nutrients and genetic material, with implications for both natural and cultivated systems (5–7). Most of these movements are seasonal and enable exploitation of favorable habitats, avoidance of unfavorable conditions, and colonization of new environments (8, 9). Understanding these insect migratory patterns is essential for integrated pest management (IPM) by improving predictions of pest outbreaks and risk of pest resistance in insect resistance management (IRM) programs.

It has long been recognized that several insect pests in North America undertake seasonal northward migrations in early spring, coinciding with favorable climatic conditions and the onset of crop production in northern regions. The so-called “pied piper” effect suggests that populations spread northward each summer but are unable to overwinter in temperate regions, as cold weather eliminates them at higher latitudes (10) source-sink dynamic: southern regions act as stable sources and northern regions function as seasonal sinks that must be repopulated annually. However, one might argue that the absence of consistent return migration, repeated northward dispersal could theoretically lead to a gradual reduction of migratory traits (11–13). Nonetheless, such traits may be preserved if migration-related behaviors are influenced by phenotypic plasticity, polygenic inheritance, or epistatic interactions among loci that maintain adaptive variation across generations (14, 15). Therefore, a reverse migration for these migratory pests should be viewed as a complementary process to the “pied piper” hypothesis (11).

*Helicoverpa zea* (Boddie) (Lepidoptera: Noctuidae), an economic pest of several crops in North America, engages in seasonal migrations, with its annual northward migration from overwintering sites in the southern United States and Mexico well documented (7, 16–18). After completing development, late-season larvae typically pupate in the soil, where survival is strongly influenced by temperature and moisture conditions (19). In northern regions, extended periods of cold prevent successful overwintering, as pupae do not survive harsh winter conditions. The occurrence of *H. zea* in northern regions requires the southward movement of adults before freezing conditions occur. Although relatively few studies have provided evidence contesting the “pied piper” effect and occurrence of late-season southward (reverse) migration (7, 20–22), its occurrence has important implications for IRM programs. Transgenic Bt traits that express toxins from the bacterium *Bacillus thuringiensis* (Cry1Ab/Ac, Cry2Ab, Cry1F, Vip3A) used in both Bt corn and Bt cotton are largely the same, leading to similar commercial trait packages adopted to manage *H. zea*. The same situation is for insecticides, adopted both in the Corn and Cotton Belts. One scenario is the possible movement of alleles for resistance in *H. zea* population, selected in the Corn Belt, being carried back to southern overwintering populations at the end of the crop season (22, 23). However, the consequences of such allele movement depend heavily on the magnitude of north-south gene flow and whether southward-moving individuals successfully produce viable offspring that contribute genetically to overwintering populations. If a substantial proportion of late-season migrants return south and reproduce, they can reinforce northern selection pressures and establish a feedback loop in which alleles selected in the Corn Belt are reintroduced into southern source populations (2). This bidirectional migratory cycle creates a feedback loop of selection pressure between northern and southern populations (11, 24), potentially accelerating the evolution of resistance in *H. zea*. An alternative scenario would be unselected populations from the crop season in northern agricultural areas cultivated with alternative non-Bt or organic crops alleviating this pressure of selection by *H. zea* moths returning to overwintering sites by the end of the crop season.

In both scenarios, the evidence of reverse migration is the first step to support modeling of risk of resistance and the development of stronger IRM programs. A useful tool for testing the hypothesis of reverse migration in *H. zea* is the use of stable hydrogen isotopes. Because hydrogen isotope values in metabolically inert tissues such as wings reflect the water sources consumed during larval development, isotopic signatures are particularly useful when adults are captured far from their emergence sites, allowing stronger inference of natal origins across broad geographic scales (7, 25–27). Previously, the use of hydrogen isotopes provided evidence of reverse migration in *H. zea* Paula-Moraes et al. (7) found that 15% of moths captured in Puerto Rico exhibited isotopic signatures consistent with origins at higher latitudes, indicating southward movement from the continental U.S. Similarly, reverse migration has been documented in fall armyworm (*Spodoptera frugiperda*) using this same isotopic approach (27), reinforcing the use of the stable hydrogen isotopes for tracking long-distance migratory movements of agricultural pests.

The present study aimed to determine the natal origins of adult *H. zea* collected across Florida, U.S. using hydrogen isotopes as biogeochemical markers. We hypothesized that hydrogen isotope values would reveal a mixture of locally emerging individuals and migrants from northern regions during late-season flights. By assessing the contribution of northern populations to southern infestations, this study provides new evidence for the bidirectional migration of *H. zea* and challenges the long-standing “pied piper” hypothesis, offering critical insights for IPM and IRM programs.

## 2 Materials and methods

### 2.1 Moth collection

**Figure 1:**
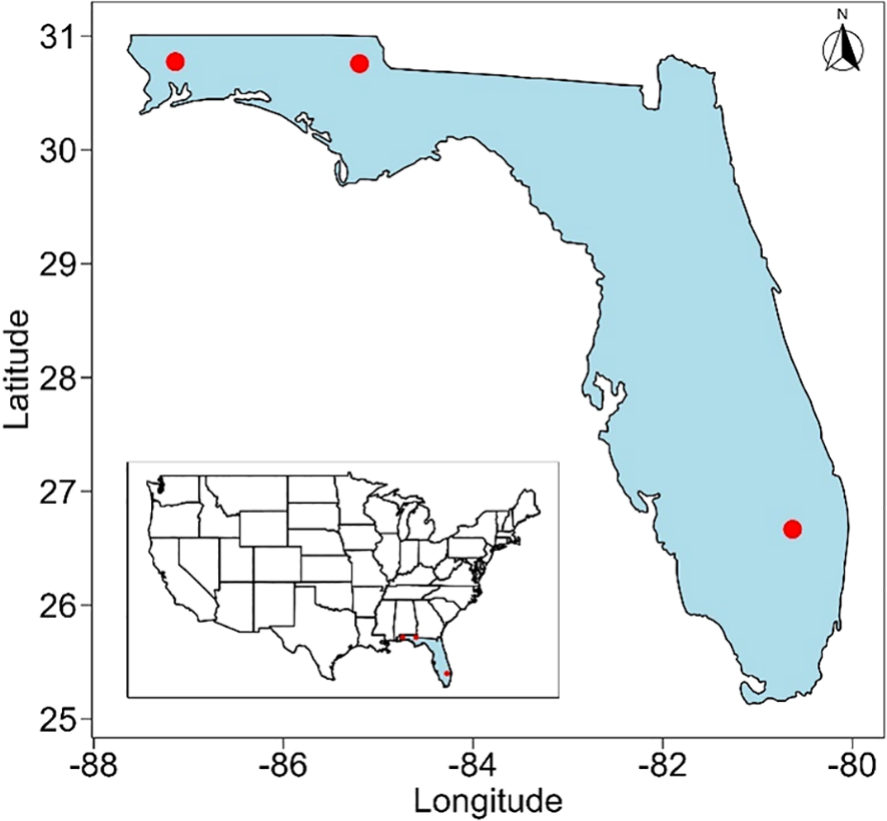
Data collection of *H. zea* moths across Florida, U.S. Red dots denote each location. Additional information about the samples is presented in **Supplementary Table 1**.

A total of 249 *H. zea* moths were collected from three regions of Florida between 2017 and 2024 (**Figure 1**). Twenty-six moths were collected in southern Florida from July to September 2024. Seventy-six moths were collected from the eastern Florida Panhandle from June to December 2017, June to November 2018, and November 2024. The remaining 147 moths were collected from the western Florida Panhandle from June to December across the years 2017 to 2024 (**Supplementary Table 1**). Collections were made using bucket traps (Hercon Luretape, Hercon Environmental, #HC-3138-10) and delta traps (Trécé, Inc., Adair, OK, U.S.) baited with a synthetic sex pheromone lure (Hercon.

Luretape, Hercon Environmental # HC-3132-10) and placed along field borders of corn, cotton, peanut, or sugarcane. Each specimen was placed individually in a 2-mL Eppendorf tube and stored at -20 °C until processing.

### 2.2 Stable hydrogen isotope analysis

The right forewing from each moth was prepared following the protocol from Calixto & Paula-Moraes (27), adapted from (26). Scales and surface oils were removed with a fine paintbrush and 70% ethanol. For specimens recovered from sticky delta trap cards, Goo and Adhesive Remover Spray Gel (Goo Gone, CC Holdings, Inc., New York, NY, U.S.) was used to dissolve adhesive residues, followed by a 24-h soak in 70% ethanol to remove any remaining contaminants. Wings were then air-dried for 24 hours, cut into fragments (0.080–0.180 mg) and stored in individual 2-mL Eppendorf tubes. This cleaning procedure has been empirically validated by Calixto & Paula-Moraes, [NO_PRINTED_FORM] (27) demonstrating effective removal of surface debris, oils, and adhesives without altering non-exchangeable δ²H values, thereby ensuring accurate isotope analysis.

Hydrogen isotope ratios were determined at the Stable Isotope Mass Spectrometry Laboratory, University of Florida, Gainesville, FL, using a Thermo Electron DeltaV Plus isotope ratio mass spectrometer with a ConFlo IV interface and high-temperature conversion elemental analyzer (TCEA). Samples and standards were sealed in 4 × 6 mm silver capsules and equilibrated for 48 h in 96-well plates before analysis (28). Non-exchangeable hydrogen values were calibrated using keratin standards CBS (Caribou Hoof Standard) and KHS (Kudu Horn Standard). Analytical precision was verified using the USGS42 standard (2.99‰; N = 11). Results were expressed in δ notation relative to Vienna Standard Mean Ocean Water (VSMOW) (**Supplementary Table 2**).

### 2.3 Natal origin assignment

The probability of origin for each sample was estimated following the approach of Ma et al. (29) using the *assignR* package in R software version 4.3.1 (30). We first constructed a hydrogen isoscape based on amount‐weighted precipitation during the growing season at 5 arc‐minute resolution. Because hydrogen isotope values in metabolically inert wing tissues reflect the larval water sources, it is important to consider whether irrigation might alter hostplant δ²H. Some corn fields on which larvae developed may have been irrigated with groundwater or reservoir water. However, hydrologic processes including groundwater recharge, soil–water mixing, and evaporation typically reduce isotopic contrasts between irrigation sources and local meteoric water, leading to partial equilibration with regional precipitation. Empirical measurements show that irrigation groundwater and local precipitation/topsoil water are isotopically very similar, differing by only about 2–3‰ in δ²H (e.g., -72‰ vs.-69.3‰), with topsoil water closely tracking these values (31). In addition, large-scale δ²H inferences minimize the potential effects of these small differences of hydrogen isotope values between different sources of water.

The isoscape was calibrated using published hydrogen isotope data included in the package database, which contains reference values from known-origin samples of the monarch butterfly (*Danaus plexippus*). Using monarch butterflies as a reference ensured consistency in taxon (Lepidoptera), geographic range, and analytical methods (32), while also providing a biologically realistic comparison for the incorporation of hydrogen isotopes into lepidopteran wing tissue. Although the calibration dataset is derived from monarch butterflies, cross-species comparisons indicate that interspecific differences in hydrogen isotope incorporation among Lepidoptera are modest. Both monarchs and *H. zea* feed on vegetation whose water is primarily sourced from shallow soil moisture closely reflecting recent precipitation, minimizing the potential for plant-water isotopic offsets. Comparisons of monarch- and *H. zea*-based calibration curves show only small differences (approximately 0-30‰ across typical precipitation gradients), which are minor relative to the broad continental δ²H gradients used for geographic assignment (~100-150‰). Therefore, these potential interspecific differences in metabolism or trophic fractionation are minimal at large-scale analysis (33, 34).

A linear model was then fitted between the precipitation isoscape and isotope values of the monarch wing tissue to generate the calibrated isoscape. Based on this calibrated surface, we applied a Bayesian inversion framework to produce posterior probability maps for each unknown origin (field-collected) sample. These maps estimate, for every grid cell, the relative probability that it represents the true origin of a given sample. From the probability surfaces, we further derived mean distance and direction metrics of potential origins. By combining these results with observed hydrogen isotope ratios, collection dates, moth behavior, and landscape context, we identified individuals that were most likely to have migrated from northern regions.

## 3 Results

**Figure 2:**
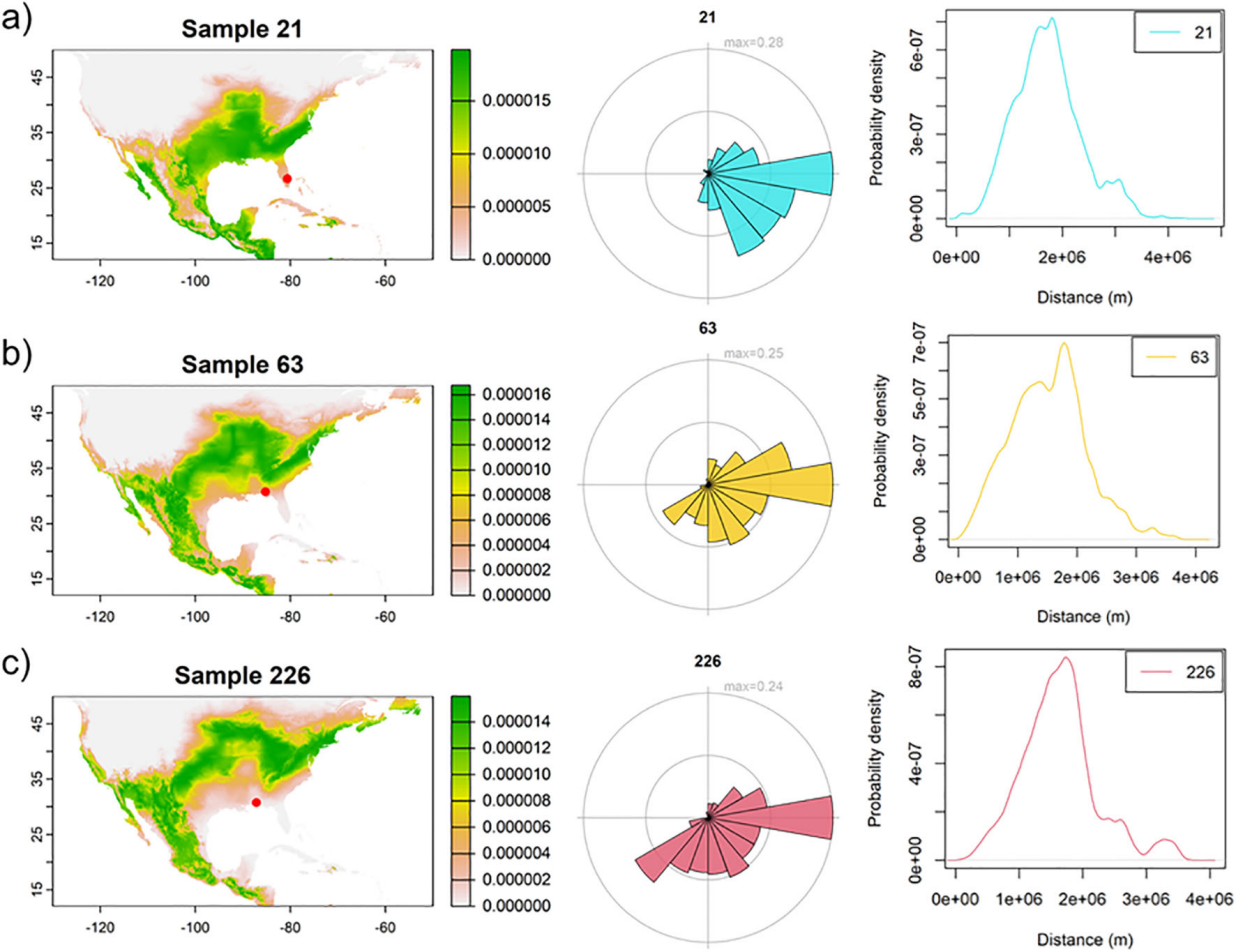
Evidence of reverse migration based on hydrogen isotope ratios in *H. zea* moths collected in **(a)** southern Florida, **(b)** eastern Florida Panhandle, and **(c)** western Florida Panhandle. Left panel: probability map with green indicating a higher probability of origin of collected moths (red dots). The Middle and Right panels represent the potential dispersal bear and distance, respectively.

**Figure 3:**
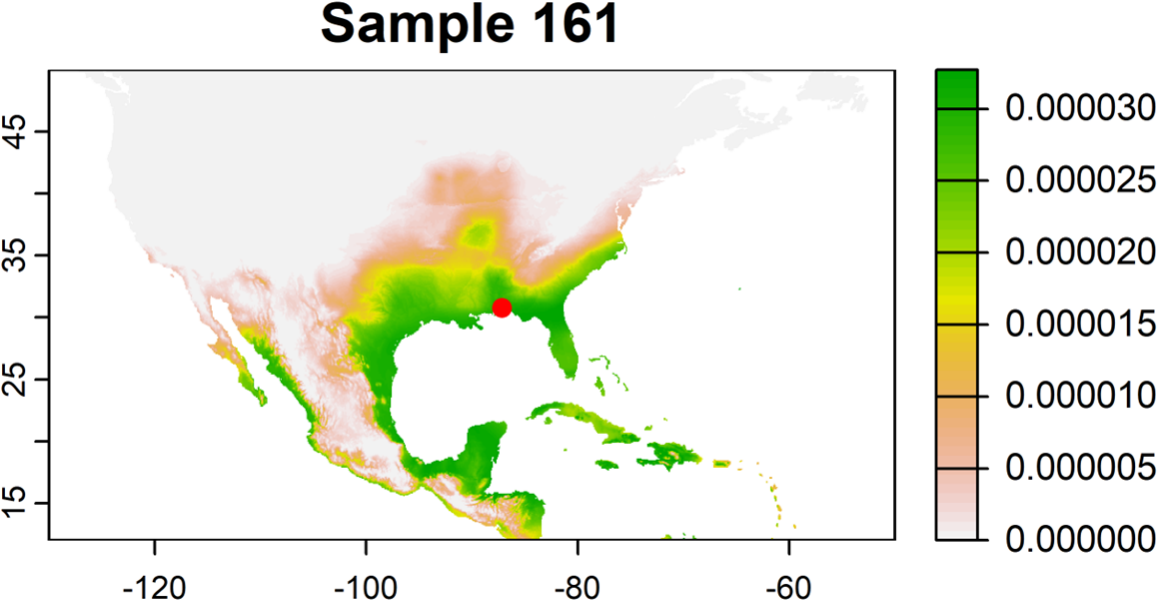
Map of the posterior probability of origin based on hydrogen isotope analysis of a moth collected in the western Florida Panhandle (red dot), which represents the pattern for ~93% of the moths collected. Green areas represent a high probability of origin.

There was evidence of southward (reverse) migration likely occurring in late summer and fall observed in approximately 7–9 moths, with one moth collected in southern Florida (**Figure 2a**), 1–2 in the eastern Florida Panhandle (**Figure 2b**), and 5–6 in the western Florida Panhandle (**Figure 2c**). Average bearing of potential sources ranged from 108° to 138°, indicating consistent northwest to southeast orientation (**Figure 2**). The probability-weighted mean distance to potential sources by the moths ranged from approximately 1,375 to 1,725 km (**Figure 2**). These results indicate a likely migration route from regions such as north Texas and Oklahoma toward the upper Midwest and Corn Belt, with additional probability of origins in the northeastern United States (**Table 1**; **Figure 2**). Posterior probability maps also assigned some individuals to regions in the western United States and Mexico. Most of the moths collected (~93%) showed a high probability of origin in Florida, Texas, and the Caribbean region (**Figure 3**).

**Table 1:** Collection regions, sampling periods, and high-probability origin areas for *Helicoverpa zea* moths collected in Florida showing potential southward (reverse) migration.

Florida collection regions	Sampling period	High-probability origin regions/states
Southern Florida	August – September 2024	Texas, Caribbean, Oklahoma, Upper Midwest/Corn Belt, Northeastern U.S.
Eastern Florida Panhandle	August – December 2017; August – November 2018; November 2024	Caribbean, north Texas, Oklahoma, upper Midwest/Corn Belt, Northeastern U.S.
Western Florida Panhandle	August – December 2017 – 2024	Caribbean, north Texas, Oklahoma, upper Midwest/Corn Belt, Northeastern U.S.

## 4 Discussion

This study provides evidence of a southward (reverse) migration of *H. zea* during late summer and fall, corresponding to the end of the crop season in the Corn Belt and supports the hypothesis of a bidirectional migratory system suggested in previous studies (7, 18, 21, 22). For instance, Paula-Moraes et al. (7), documented the first direct evidence of reverse migration using hydrogen isotopic markers, indicating a southward movement of late-season populations from northern regions in the U.S and South of Canada. Building on this broader evidence, our Florida-focused analysis detected a subset of moths with isotope values consistent with northern origins, demonstrating that reverse migrants reach multiple regions within the state. The results suggest long-distance southward movement from regions such as north Texas, Oklahoma, and the upper Midwest, particularly the Corn Belt. While most individuals were assigned to origins in Florida, Texas, and the Caribbean, the presence of migrants from northern populations suggests that local infestations late in the season may be reinforced by long-range dispersal. Although some posterior probability maps indicated additional high-probability origin areas in the western United States and Mexico, we consider these regions unlikely true sources for the moths collected in Florida. Stable isotope assignments can converge on isotopically similar regions that are not connected by realistic migratory pathways, particularly where prevailing winds and major topographic barriers constrain long-distance eastward movement. Consistent with previous isotope-based migration studies showing that *H. zea* and *S. frugiperda* primarily use wind-assisted seasonal corridors linking the Corn Belt, southeastern United States, Gulf Coast, and Caribbean (7, 27), origins in regions such as Texas, Oklahoma, and the Midwestern United States are more plausible than western U.S. or Mexican source.

Seasonal movements appear to function as an adaptive strategy for population persistence, enabling *H. zea* to reduce exposure to harsh winter conditions in northern areas by shifting to southern regions with milder climates where they can overwinter. These findings are consistence with previous work suggesting that *H. zea* has limited capacity to survive winters in colder northern regions (19) and must annually recolonize these areas each spring and summer via seasonal migration (7, 33–35). Comparable overwintering limitations have been documented in other noctuid species, such as *S. frugiperda*, which rely on southern refugia, including Florida, Texas, and northern Mexico, to persist through winter (27, 36–38). For *H. zea*, successful overwintering is primarily restricted to USDA plant hardiness zones 8b and higher, where soil temperatures remain above lethal thresholds (33, 39, 40). This limitation arises from its soil-pupating habit, where larvae typically burrow 2–10 cm below the surface to pupate (34, 41, 42). However, shallow burial leaves pupae exposed to fluctuating soil conditions, and prolonged exposure to subfreezing temperatures (≤ –5 °C) at these depths results in near-complete mortality (43). Consequently, pupae cannot persist through winters in northern latitudes where soils regularly freeze, restricting survival to southern zones with frost-free or minimally freezing winters. Importantly, these overwintering constraints do not by themselves imply that a consistent southward return migration is required for long-term population persistence. Annual recolonization of northern regions from southern overwintering sources remains a well-supported mechanism, and source-sink dynamics can fully sustain population connectivity and gene flow without necessitating a large-scale reverse migration event. Our detection of isotopic signatures consistent with northern origins should therefore be viewed as evidence of a possible complementary process rather than a prerequisite for population viability (18).

Our findings suggest that the northward annual migration expansion of *H. zea* is followed by a seasonal retreat to southern overwintering areas due to these barriers for pupal survival. In addition, our results suggest that during the late season, populations of *H. zea* collected in the Florida Panhandle and in the peninsula are a mixture of locally emerging moths and migrants from northern areas, supporting the bidirectional migration dynamics of *H. zea* (7, 16, 18, 21, 22). Although we document reverse migration in a subset of individuals, these results do not imply that long-term persistence of *H. zea* requires a consistent southward return migration. Recolonization of northern habitats each spring from southern overwintering refugia remains a biologically robust and ecologically supported mechanism for population maintenance. Many migratory insects operate under source-sink dynamics in which southern regions act as stable sources and northern areas function as seasonal sinks that must be repopulated annually. Under this framework, retention of migratory traits can arise through behavioral plasticity, broad genetic architecture, or repeated annual recolonization rather than requiring a strict bidirectional cycle. Thus, our findings support reverse migration as a complementary process rather than the exclusive alternative to the pied-piper hypothesis within the broader seasonal dynamics of *H. zea* (7, 11, 12, 19).

Similar patterns of seasonal population connectivity in *H. zea* have been inferred by Perera et al. (2) using single nucleotide polymorphism (SNP) analysis demonstrating high levels of gene flow and regional dispersal of this species across the southeastern United States. Their findings suggested that populations are not isolated but instead experience frequent genetic exchange, consistent with the large-scale seasonal movements inferred from hydrogen isotopic data generated in our study. Legan et al. (44) have also shown extensive gene flow among *H. zea* field populations across multiple southern states in the U.S. based on genome-wide SNP frequencies. Their analysis revealed minimal genetic differentiation across populations sampled from diverse field sites, supporting the concept of a well-mixed, highly mobile pest population (44). This connectivity is expected to accelerate the risk of spreading alleles, including those conferring resistance to Bt toxins and insecticides.

The evidence presented here aligns closely with recent findings on *S. frugiperda*. Using the same hydrogen isotopic markers, Calixto & Paula-Moraes (27), documented reverse migration in a subset of moths collected in Florida, with high probability of origin in northern Corn Belt, highlighting Florida and the broader Gulf Coast as mixing zones where northern migrants and southern overwintering populations converge (27). This southward movement may not be unique to *H. zea* and *S. frugiperda* and could also occur in other major migratory noctuid pests. For instance, *Anticarsia gemmatalis* (45), *Trichoplusia ni* (46), *Chrysodeixis includens* (47), and *S. exigua* (48) are known to spread northward annually in the eastern United States and breed in regions unsuitable for overwintering (15). The parallels among *H. zea* and *S. frugiperda* suggest that reverse migration is a shared life-history strategy of noctuid pests, enabling persistence across latitudinal gradients while maintaining genetic connectivity on a continental scale (7, 16).

Reverse migration in *H. zea* not only ensures overwintering survival but also creates pathways for the long-distance movement of resistance alleles across North America, with potential impact for management measures. Insecticides and commercial Bt traits are largely the same adopted in Corn and Cotton belts, increasing the risk of accelerating the spread of resistance traits across regions and should be taken in consideration in IPM and IRM programs. Populations of *H. zea* from the Corn Belt are exposed to selection pressure on Bt corn and insecticides and consequently act as sources of resistance alleles, which are subsequently transported southward during late-season migrations to the Cotton Belt. If resistance alleles continue to be selected in the Bt corn in the Corn Belt, the southward movement of these alleles have the potential to accelerate the spread of resistance into overwintering populations in the Cotton Belt, thereby magnifying the challenge of managing *H. zea*. In 2024, approximately 86% of U.S. corn and 90% of cotton acreage were planted with Bt (49, 50). Over the past two decades, Bt adoption has steadily increased, with stacked traits now dominating production, creating extensive and nearly continuous exposure of *H. zea* to Bt toxins.

In summary, our study strengthens the evidence of reverse migration in *H. zea*, which should be considered when evaluating population dynamics, gene flow, and resistance risk across North America. This southward movement facilitates overwintering and continue population survival while enabling the long-distance transport of resistance alleles. Northern populations exposed to strong selection pressures from Bt crops and insecticides in the Corn Belt is expected to act as sources of resistance alleles that are subsequently redistributed southward during late-season migrations (23, 51). Such seasonal connectivity promotes the continental-scale spreading of resistance, underscoring the complexity of managing this highly mobile pest. Future research should incorporate stable isotope markers, advanced population genomics, and migration trajectory modeling to quantify the frequency, environmental drivers, and ecological consequences of southward migration events and the risk of evolution of resistance in *H. zea* to insecticides and Bt technology. Such multidisciplinary approaches are urgently needed for improving IPM and IRM programs, thereby increasing the long-term durability of management measures in the diverse agricultural landscapes of North America.

## Data Availability

The original contributions presented in the study are included in the article/**Supplementary Material**. Further inquiries can be directed to the corresponding author.
