# Supplementary material for: Challenging the long-held “pied piper” hypothesis: evidence of southward migration of corn earworm (Helicoverpa zea) in North America
Source: Front Insect Sci. 2026 Apr 24;6:1726127. doi: 10.3389/finsc.2026.1726127 (PMC13152866; doi:10.3389/finsc.2026.1726127)
Supplement: Supplementary file 1 [file Table1.docx]

| **Location** | **Latitude (°N)** | **Longitude (°W)** | **Year** | **Sampling Period** | **Total Samples** |
| --- | --- | --- | --- | --- | --- |
| South Florida | 26.6665 | -80.6325 | 2024 | July - September | 26 |
| East Florida Panhandle | 30.7644 | -85.2554 | 2017 | June - December | 20 |
|  |  |  | 2018 | June - September, Nov | 49 |
|  |  |  | 2024 | November | 7 |
| West Florida Panhandle | 30.7644 | -87.1393 | 2017 | July - December | 14 |
|  |  |  | 2018 | June - December | 64 |
|  |  |  | 2019 | October | 10 |
|  |  |  | 2020 | October - November | 10 |
|  |  |  | 2022 | October | 2 |
|  |  |  | 2023 | October - December | 15 |
|  |  |  | 2024 | October - November | 3 |

**Table 1**:Total number of Helicoverpa zea moths collected in south Florida, and in east and west Florida Panhandle
